# The Aurora B-controlled PP1/RepoMan complex determines the spatial and temporal distribution of mitotic H2B S6 phosphorylation

**DOI:** 10.1098/rsob.230460

**Published:** 2024-05-29

**Authors:** Maximilian Pfisterer, Roman Robert, Vera V. Saul, Amelie Pritz, Markus Seibert, Regina Feederle, M. Lienhard Schmitz

**Affiliations:** ^1^ Institute of Biochemistry, Justus-Liebig-University Giessen, Giessen, Germany; ^2^ Monoclonal Antibody Core Facility, Helmholtz Center Munich, German Research Center for Environmental Health, Neuherberg, Germany

**Keywords:** mitosis, histone phosphorylation, phosphatase scaffold, centromere

## Abstract

The precise spatial and temporal control of histone phosphorylations is important for the ordered progression through the different phases of mitosis. The phosphorylation of H2B at S6 (H2B S6ph), which is crucial for chromosome segregation, reaches its maximum level during metaphase and is limited to the inner centromere. We discovered that the temporal and spatial regulation of this modification, as well as its intensity, are governed by the scaffold protein RepoMan and its associated catalytically active phosphatases, PP1α and PP1γ. Phosphatase activity is inhibited at the area of maximal H2B S6 phosphorylation at the inner centromere by site-specific Aurora B-mediated inactivation of the PP1/RepoMan complex. The motor protein Mklp2 contributes to the relocalization of Aurora B from chromatin to the mitotic spindle during anaphase, thus alleviating Aurora B-dependent repression of the PP1/RepoMan complex and enabling dephosphorylation of H2B S6. Accordingly, dysregulation of Mklp2 levels, as commonly observed in tumour cells, leads to the lack of H2B S6 dephosphorylation during early anaphase, which might contribute to chromosomal instability.

## 1. Introduction

All phases of mitosis are accompanied by massive changes in post-translational modifications (PTMs) [1,2]. In this context, changes in phosphorylations make an essential contribution to the temporally coordinated control of the different phases of chromosome condensation, spindle assembly, sister chromatid segregation and cytokinesis [3,4]. Among other proteins, these transient phosphorylations are also found on histones including H2A S1, H2B S6, H3 S10, H3 T3, H4 S1, linker histones and histone variants including the histone protein H3 variant CENP-A (centromere protein A) [4]. A characteristic feature of most of these transient mitotic phosphorylations is their highly specific temporal and spatial distribution. While some mitotic phosphorylations are restricted to the inner centromere (H2B S6 and H3 T3), others occur at the kinetochore-proximal centromere (CENP-A S7, H2A T120), whereas phosphorylations at H3 S10 and H3 S28 are found on chromosome arms [5]. The temporal order and duration of individual phosphorylations are intricately regulated to precisely match the specific function of each phosphorylation. This regulation is achieved by precise timing of recruitment, activation and control of protein kinases and their antagonizing phosphatases. In addition, some phosphorylation sites such as H3 S10 are modified by multiple kinases to increase the redundancy and the spectrum of input signals [6–9]. One of the H3 S10 modifying kinases is Aurora B, which is part of the chromosomal passenger complex (CPC) [10]. In addition to Aurora B, this multi-protein complex also contains adapter proteins controlling the localization and activity of the assembly, namely, Survivin and Borealin and the inner centromere protein (INCENP) [10]. Borealin, Survivin and the N-terminal domain of INCENP mediate centromere targeting of the CPC to allow locus-specific phosphorylation [11–13].

Also, the activity and localization of phosphatases is controlled by differentially composed multi-protein complexes [14,15]. The protein phosphatase 1 (PP1) family of serine/threonine phosphatases can interact with more than 200 regulatory proteins and is involved in the dephosphorylation of a wide range of protein substrates during mitosis, meiosis and other processes [16]. PP1 enzymes are typically composed of one or more regulatory subunits and a catalytic subunit. Mammals express four PP1 catalytic subunits, namely PP1α, PP1β and PP1γ1, as well as the testis-specific PP1γ2 [17]. Regulatory proteins localize PP1 to specific regions and guide the catalytic subunits to their cognate substrates [18,19]. Some of the targeting subunits have a known function during the late stages of mitosis, namely, RepoMan (recruits PP1 onto mitotic chromatin at anaphase), Ki-67 and PNUTS (PP1 nuclear targeting subunit) [20–22]. The association of RepoMan with PP1 subunits is dynamic and controlled by regulatory phosphorylations mediated by Aurora B or CDK1/cyclin B [23–26]. As the counterpart to mitotic kinases, these catalytic and regulatory PP1 subunits are of great importance for the correct timing of substrate dephosphorylation and chromosome segregation [27–30]. Consequently, misregulated histone phosphorylation can lead to the appearance of chromosomal instability (CIN), which is the characteristic of many tumour cells [31].

Recent progress in biochemical and computational approaches has drastically expanded our knowledge on the different kinases responsible for adding phosphate groups to their respective substrates [32,33]. By contrast, our knowledge of phosphorylation erasers is limited owing to the highly complex subunit composition of the subunits of phosphatase complexes.

We have previously discovered H2B S6ph as a new histone modification with a functional role in mitosis and also identified the writer (CDK1/cyclin B) and reader (SET) [34]. Here, we set out to identify the eraser and the molecular mechanisms by which it controls the spatiotemporal distribution of H2B S6ph. We found that PP1α and PP1γ can independently dephosphorylate H2B S6ph at the onset of anaphase. Both phosphatases can associate with the scaffold protein RepoMan, which is phosphorylated by centromeric Aurora B to interfere with chromatin binding and association with the catalytic PP1 subunits. Proper dephosphorylation of H2B S6 during early anaphase depends on Mklp2-driven relocalization of Aurora B from chromatin to the central spindle, alleviating repression of PP1/RepoMan.

## 2. Results

### 2.1. H2B S6 is dephosphorylated by PP1α and PP1γ

**Figure 1.**
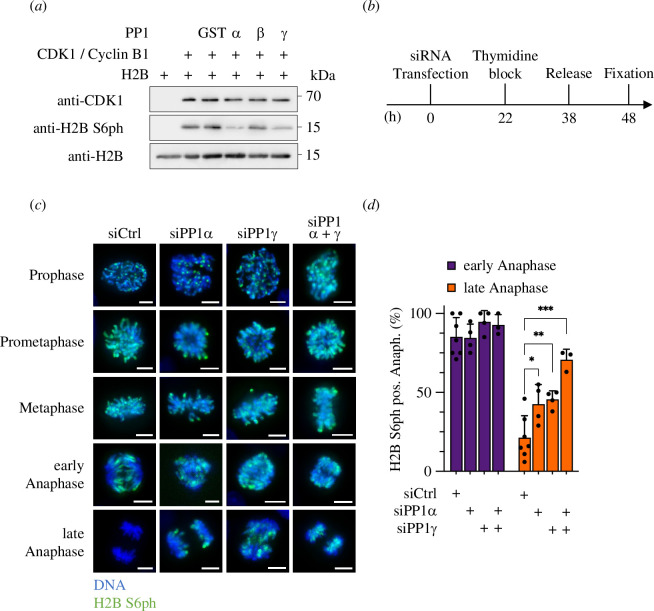
Identification of the PP1 subunits mediating dephosphorylation of H2B S6ph. (*a*) Purified H2B protein was phosphorylated by CDK1 and cyclin B *in vitro* to allow for H2B S6ph. CDK1/cyclin B was inactivated by heating and 0.2 µg of recombinant PP1 subunits or glutathion-S-transferase (GST) as a control were added. After further incubation for 30 min, proteins were analysed by Western blotting with the indicated antibodies, the positions of molecular weight markers are indicated. (*b,c*) HCT116 cells were treated as schematically indicated in (*b*) and analysed for the occurrence of H2B S6ph during the various mitotic stages as shown (*c*), scale bar = 10 µm. (*d*) The occurrence of H2B S6ph during early and late anaphase detected in (*c*) was quantified and statistically analysed with a two-way ANOVA and Tukey multiple comparisons correction from more than three independent biological replicates with *n* = 40, *p  ≤  0.05, **p  ≤  0.01, ***p  ≤  0.001.

To facilitate the analysis of mitotic H2B S6ph and to enable co-staining with other mitotic regulators we generated a rat monoclonal antibody that specifically detects this histone modification. Functional analysis of different hybridomas showed that antibody clone 1D4 is suitable for Western blotting and immunofluorescence analysis and detects H2B phosphorylation at S6 with high specificity (electronic supplementary material, figures S1 and S2). Binding of the antibody to its cognate sequence is not affected by H2B K5 methylation, but impaired by H2B K5 acetylation (electronic supplementary material, figure S3*a*). Since this histone modification is not changed during mitosis (electronic supplementary material, figure S3*b*,*c*), all changes of the antibody signal can be assigned to dynamic H2B S6ph. As previous work from us has shown that H2B S6 dephosphorylation proceeds via PP1 [34], it was relevant to identify the responsible PP1 subunit(s). *In vitro* experiments were performed where recombinant H2B was phosphorylated by incubation with CDK1 and cyclin B, followed by their inactivation and the addition of purified PP1α, PP1β and PP1γ. Subsequent detection of H2B S6ph by immunoblotting revealed dephosphorylating activity for PP1α and PP1γ, but not for PP1β (figure 1*a*). The contribution of endogenous PP1α and PP1γ subunits for *in vivo* H2B S6 dephosphorylation was analysed by knockdown experiments. Diploid HCT116 cells were transfected with siRNAs targeting the different PP1 phosphatases and synchronized using a thymidine block/release protocol, as schematically shown in figure 1*b*. The analysis of mitotic cells showed H2B S6ph at the inner centromere between prophase and metaphase, spatial extension of the phosphorylation during early anaphase and absent H2B S6ph during late anaphase of control cells, as previously described [34]. The knockdown of PP1α and PP1γ either alone or in combination reduced H2B S6ph dephosphorylation, as revealed by immunofluorescence analysis and its statistical analysis (figure 1*c,d*; electronic supplementary material, figure S4). The highest percentage of H2B S6ph in late anaphase was seen after combined downregulation of PP1α and PP1γ (figure 1*d*). The additive effect of the combined knockdown of both PP1α and PP1γ suggests that both subunits are able to dephosphorylate H2B S6 independently.

**Figure 2.**
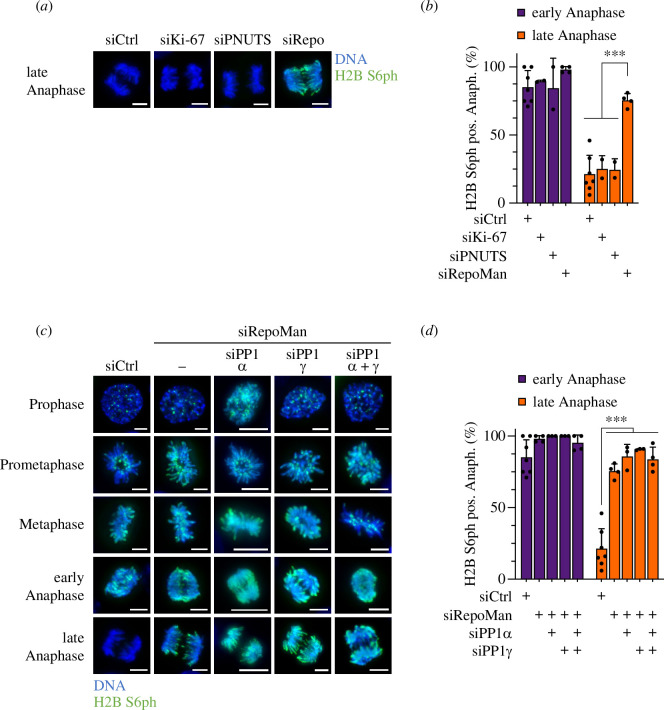
Control of temporal dynamics of mitotic H2B S6ph by RepoMan and PP1. (*a,b*) HCT116 cells were treated with siRNAs targeting the indicated PP1 scaffold proteins are further treated as schematically shown in figure 1*b*. The occurrence of H2B S6ph during late anaphase was analysed by indirect immunofluorescence (*a*) and analysed quantitatively (*b*). (*c,d*) HCT116 cells were treated with siRNAs targeting RepoMan and the PP1α and PP1γ subunits as shown, followed by analysis of mitotic H2B S6ph by indirect immunofluorescence (*c*) and its quantification (*d*). Scale bar = 10 µm, statistical analysis was done using a two-way ANOVA and Tukey multiple comparisons correction from more than three independent biological replicates with *n* = 40, ***p  ≤  0.001.

PP1 is commonly recruited to its targets via its unique interaction motif or by targeting factors. Mitotic PP1 chromatin targeting factors include the proliferation marker Ki-67, PNUTS and RepoMan [20–22]. To identify the targeting subunit controlling mitotic PP1α/γ-mediated H2B S6 dephosphorylation, further siRNA experiments interfering with the expression of these proteins were performed. The analysis of mitotic cells showed that only the downregulation of RepoMan led to defective dephosphorylation, as revealed by the occurrence of H2B S6ph in late anaphase (figure 2*a,b*; electronic supplementary material, figure S5). Further, knockdown experiments were performed by combinatorial downregulation of RepoMan together with PP1α and/or PP1γ. Downregulation of RepoMan caused reduced of H2B S6 dephosphorylation after early anaphase, but this effect was not further augmented by simultaneous knockdown of PP1α and/or PP1γ, as revealed by immunofluorescence (figure 2*c*) and its quantitative analysis (figure 2*d*). These data are consistent with the notion that RepoMan binds PP1γ and also to a significantly lesser extent PP1α [20,24], as also confirmed by pull-down experiments (electronic supplementary material, figure S6).

**Figure 3.**
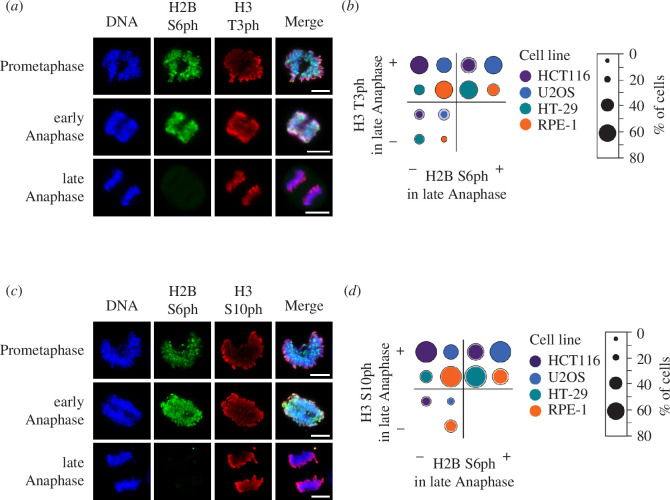
Comparative analysis of different histone phosphorylations during anaphase. (*a*) HCT116 were synchronized and analysed for H3 T3ph and H2B S6ph during anaphase as shown. (*b*) The experiment from (*a*) was performed using the indicated cell lines and statistically analysed. The diameter of the circles corresponds to the percentage frequency, the outer ring indicates the value of the upper standard deviation from three biological replicates. (*c,d*) The experiment was performed as for (*a,b*) with the difference that anaphase phosphorylation of H3 S10ph and H2B S6ph was compared, scale bar = 10 µm.

Although RepoMan plays a central role in dephosphorylating H3 S10 and H3 T3 [26,35], dephosphorylation of these sites at the inner centromere occurred with different kinetics (figure 3*a*). The quantitative analysis of the phosphorylation status at these sites in different immortalized cells and tumour cell lines during anaphase showed—consistent with previously published data—H3 T3ph in a substantial percentage of anaphase cells [35,36], while H2B S6 modification has already declined (figure 3*b*). Of note, we never observed H2B S6ph in the absence of H3 T3ph during late anaphase. Also, the comparison between H2B S6 and H3 S10 dephosphorylation during anaphase revealed distinct kinetics (figure 3*c*). H2B S6ph was never observed in the absence of H3 S10ph during late anaphase and a quantitative analysis showed that a substantial fraction of cells showed H3 S10 modification in the absence of H2B S6 phosphorylation (figure 3*d*). Together, these data indicate that despite the shared importance of the PP1/RepoMan system, the duration of histone modifications can occur with staggered timing.

### 2.2. RepoMan controls the restriction of H2B S6ph to the inner centromere

**Figure 4.**
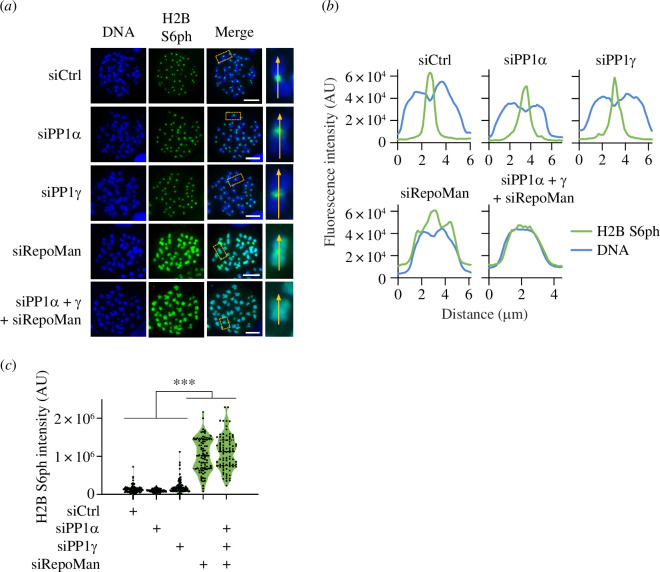
Control of spatial distribution and intensity of mitotic H2B S6ph by RepoMan and PP1. (*a*) HCT116 cells were treated with the indicated siRNAs and then arrested at metaphase using nocodazole. Chromosome spreads were analysed for chromosomal localization of H2B S6ph. The boxed areas are shown in higher magnification, the arrows indicate the chromosome axis, scale bar = 10 µm. (*b*) Line scan analysis for the distribution of H2B S6ph and DNA along the chromosome axis of the chromosomes shown in (*a*). (*c*) H2B S6ph detected on the chromosome spreads displayed in (*a*) was quantified using ImageJ and statistically analysed with a one-way ANOVA and Dunnett multiple comparisons correction with *n* = 87, ***p  ≤  0.001.

While the experiments performed so far show a function of PP1/RepoMan for the temporal control of H2B S6 phosphorylation, it was relevant to investigate its potential contribution to the spatial restriction of this modification. To address this question, HCT116 cells were transfected with siRNAs specifically targeting PP1α, PP1γ or RepoMan. Cells were arrested at prometaphase by a nocodazole block and chromosomal spreads were stained for the occurrence and chromosomal distribution of H2B S6ph. While knockdown of the catalytic PP1 subunits alone did not affect the restriction of H2B S6ph to the inner centromere, interference with RepoMan resulted in spreading of this modification along the entire chromosome, as revealed by immunofluorescence (figure 4*a*) and its quantitative analysis (figure 4*b*). Downregulation of RepoMan either alone or in combination with PP1α and PP1γ not only showed a spatial change in H2B S6 phosphorylation but also caused an increase in the intensity of this histone modification (figure 4*c*). Together, these experiments reveal the central importance of the RepoMan phosphatase scaffold for the control of the intensity as well as the temporal and spatial distribution of H2B S6ph.

### 2.3. Aurora B-mediated RepoMan modification mediates temporal control of H2B S6 dephosphorylation

**Figure 5.**
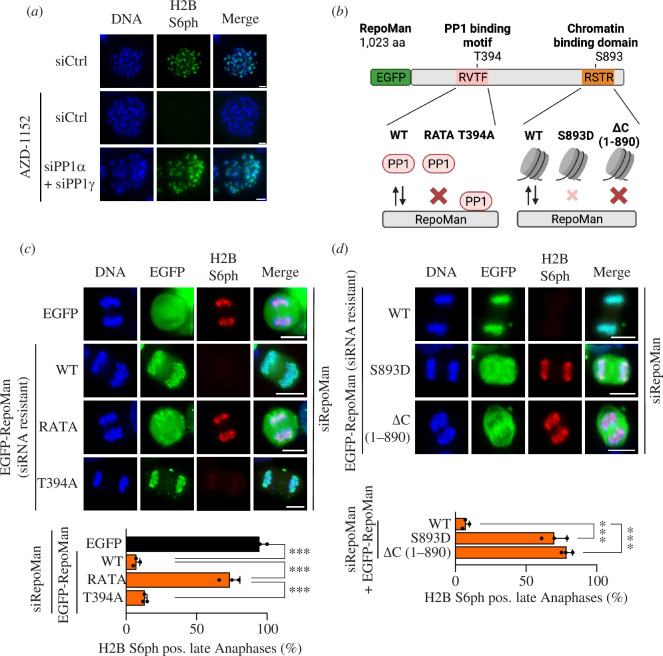
Control of mitotic H2B S6ph by RepoMan phosphorylation. (*a*) HCT116 cells were transfected with the indicated siRNAs and then treated with nocodazole and the Aurora B inhibitor AZD-1152 (1 µM). Chromosome spreads were prepared from metaphase cells and H2B S6ph was detected by immunofluorescence as shown. (*b*) Schematic representation of the RepoMan protein and the effects of phosphosite mutations on binding to chromatin and PP1. (*c*) HCT116 cells were transfected with an siRNA targeting the endogenous RepoMan mRNA together with plasmids directing the expression of siRNA-resistant enhanced green fluorescent protein (EGFP)-RepoMan mutants or an EGFP control as indicated. H2B S6ph and EGFP expression were detected by immunofluorescence (upper) and quantified (lower). Statistical analysis was done with a one-way ANOVA and Dunnett multiple comparisons correction from three biological replicates with *n* = 30. (*d*) The experiment was performed as in (*c*) with the difference that RepoMan mutants compromising the chromatin association were used. Scale bar = 10 µm, ***p  ≤  0.001.

We have previously observed that H2B S6ph is dependent on Aurora B kinase activity [34], but the underlying molecular mechanism is not known. To investigate whether this inhibition depends on PP1, HCT116 cells were treated with siRNAs targeting PP1α and PP1γ or adequate controls, followed by interference with Aurora B kinase activity using the specific inhibitor AZD-1152 [37]. The analysis of H2B S6ph on chromosomal spreads from nocodazole-treated cells showed that absent phosphorylation in the presence of AZD-1152 was fully rescued upon downregulation of PP1α/γ (figure 5*a*), revealing that Aurora B restricts H2B S6ph exclusively by a PP1-dependent mechanism. Previous work has shown that Aurora B-mediated phosphorylation of RepoMan at S893 impairs its chromatin association, while phosphorylation at T394 and probably further sites in its vicinity disables PP1 association [23–25].

To investigate the potential contribution of these RepoMan modifications for the control of H2B S6ph, various RepoMan mutants affecting binding to PP1 (T394A and RATA) or disabling RepoMan’s ability to associate with chromatin (S893D and RepoMan ΔC (1-890)), were created, as schematically shown in figure 5*b*. To investigate the contribution of RepoMan’s PP1 binding capability for H2B S6 dephosphorylation, HCT116 cells were treated with a RepoMan siRNA and transfected with siRNA-resistant forms of RepoMan, RepoMan T394A and RepoMan RATA. The analysis of H2B S6ph in late anaphase showed that expression of RepoMan WT or the RepoMan T394A mutant (leading to increased PP1 association) resulted in absent H2B S6ph in late anaphase. By contrast, expression of the RepoMan RATA mutant (abolishing PP1 binding) resulted in defective dephosphorylation (figure 5*c*). Similarly, impaired (RepoMan S893D) or absent (RepoMan ΔC 1-890) chromatin association of this phosphatase scaffold protein reduced H2B S6 dephosphorylation in late anaphase (figure 5*d*; electronic supplementary material, figure S7). Collectively, these data suggest that centromere-associated Aurora B modifies RepoMan to prevent its ability to associate with chromatin and PP1, thus preserving centromeric H2B S6ph. It will be interesting to study in the future also whether CDK1-mediated phosphorylations of RepoMan contribute to the control of H2B S6 dephosphorylation.

### 2.4. Aurora B mediates spatial control of H2B S6ph

**Figure 6.**
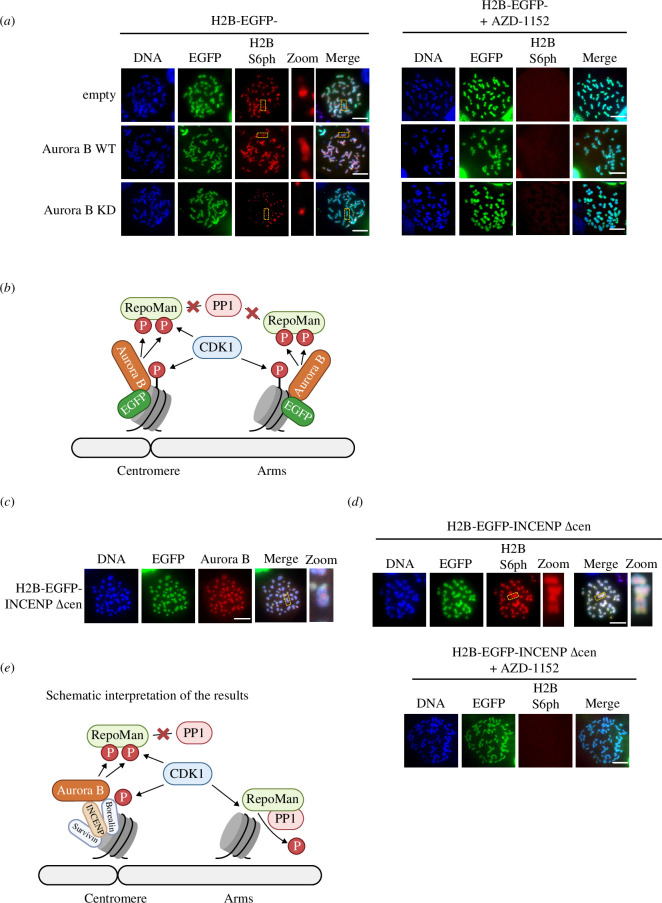
Analysis of Aurora B-mediated RepoMan inactivation. (*a*) Left: HCT116 cells were expressed to express H2B-EGFP-Aurora B, a kinase-inactive point mutant thereof (H2B-EGFP-Aurora B KD) or H2B-EGFP (empty) as a control. Chromosome spreads from nocodazole-arrested cells were stained for localization of the EGFP-tagged fusion protein and H2B S6ph, the boxed areas are shown in higher magnification. Right: the experiment was done as in the left part with the difference that AZD-1152 (1 µM) and nocodazole were added simultaneously. (*b*) The H2B-EGFP-Aurora B fusion protein is incorporated along the entire chromosome. The resulting inhibition of phosphatase leads to the spreading of H2B S6ph along the entire chromosome. (*c*) HCT116 cells were transfected to express H2B-EGFP-INCENP Δcen. Chromosome spreads of nocodazole-arrested cells were stained to reveal the localization of the EGFP fusion protein and Aurora B. (*d*) H2B-EGFP-INCENP Δcen was expressed in HCT116 cells and cells were treated with nocodazole either alone or in combination with AZD-1152. Chromosome spreads from mitotic cells were stained for spatial distribution of H2B-EGFP-INCENP Δcen and H2B S6ph as shown, scale bar = 10 µm. (*e*) Schematic interpretation of results. While the constitutive CDK1/cyclin B-mediated H2B S6ph occurs along the entire chromosome, the antagonizing phosphatase is inactivated specifically at the centromere. This occurs through centromere-specific and CENP-A-mediated anchoring of INCENP and its associated Aurora B kinase.

To further study a potential contribution of Aurora B to the spatial control of H2B S6ph, this kinase was fused to a H2B-EGFP protein to allow its tethering along the entire chromosome. Expression of these fusion proteins led to the spreading of H2B S6ph along the chromosome arms, as revealed by the analysis of chromosome spreads from mitotic cells (figure 6*a*; electronic supplementary material, figure S8*a*) and their quantitative analysis (electronic supplementary material, figure S8*b*). Expression of a kinase-inactive H2B-EGFP-Aurora B point mutant (H2B-EGFP-Aurora B KD) did not allow spreading of the H2B S6 phosphorylation, revealing the relevance of Aurora B kinase activity for this event. The remaining H2B S6 phosphorylation at the inner centromere in the presence of H2B-EGFP-Aurora B KD is probably attributable to the activity of the endogenous kinase, as suggested by completely absent phosphorylation in the presence of the specific Aurora B inhibitor AZD-1152 (figure 6*a*). These data suggest that Aurora B activity is necessary and sufficient to protect H2B S6ph from dephosphorylation by PP1/RepoMan. Inappropriate localization of Aurora B to chromosome arms leads to the spreading of H2B S6ph, as schematically displayed in figure 6*b*.

To substantiate these findings by an independent experimental approach, we interfered with centromere-specific localization of Aurora B upon expression of H2B-EGFP-INCENP ∆cen, a mutant lacking its centromeric binding domain (aa 1–46) [11]. While some Aurora B remained attached to the centromere owing to the presence of the endogenous INCENP wild type (WT) protein, cells expressing H2B-EGFP-INCENP Δcen showed extended localization of the truncated INCENP and of Aurora B on the chromosome arms (figure 6*c*; electronic supplementary material, figure S9). Also in this model, non-centromeric localization of Aurora B led to the spreading of H2B S6 phosphorylation along the chromosome arms. This effect was again fully dependent on the kinase activity of Aurora B (figure 6*d*), corroborating the key role of Aurora B kinase activity for the spatial control of H2B S6ph. Together, these results suggest a model where Aurora B kinase activity protects H2B S6ph from dephosphorylation by PP1/RepoMan specifically at the inner centromere, while constitutive CDK1-mediated phosphorylation at the chromosome arms is immediately antagonized by PP1/RepoMan (figure 6*e*).

### 2.5. Mklp2-controlled mislocalization of Aurora B contributes to aberrant H2B S6ph in tumour cells

**Figure 7.**
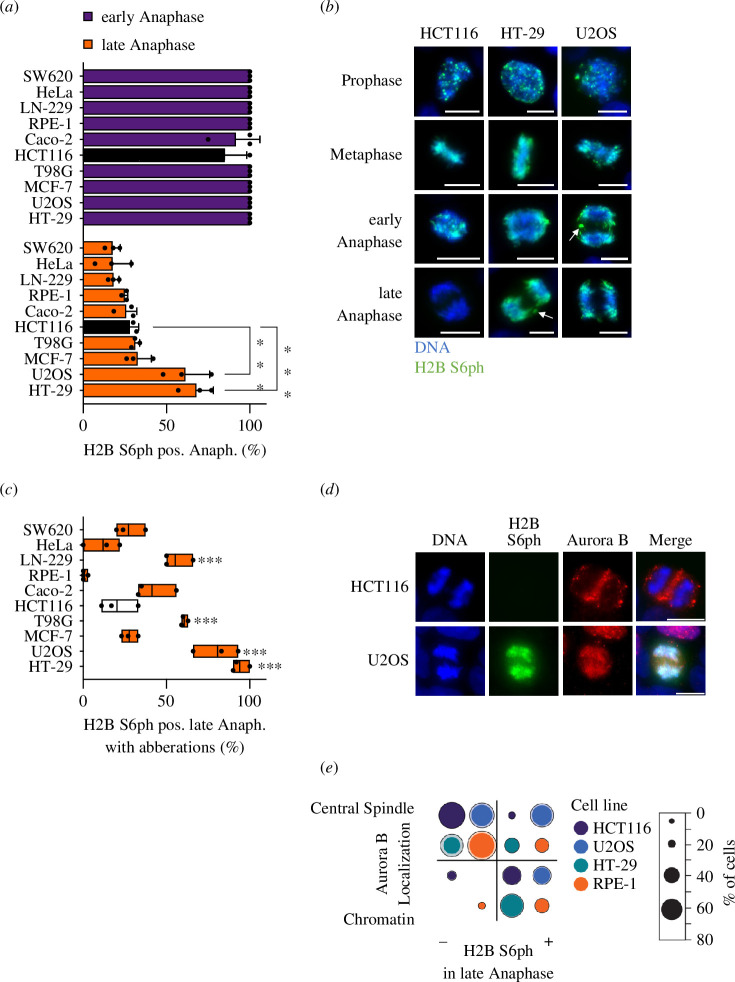
Distribution of H2B S6ph in tumour cells with CIN. (*a*) The indicated cells ranging between high (HCT116, RPE-1) and low (U2OS, HT-29) genetic stability were analysed for the occurrence of H2B S6ph during early or late anaphase. Statistical analysis was performed with two-way ANOVA and Tukey correction for multiple comparisons from three biological replicates with *n* = 40. HCT116 cells are indicated by black bars, as they were used in all previous experiments of this study. (*b*) H2B S6ph was detected during the different mitotic phases in the indicated cell lines, arrows point to enriched H2B S6ph in chromosomal aberrations. (*c*) Chromosomal aberrations showing H2B S6ph in late anaphase were analysed in the indicated cell lines. One-way ANOVA with Dunnett correction for multiple comparisons was performed from three biological replicates with *n* = 25. All cell lines were compared with HCT116 cells, indicated by a white box. Box plots show the data between the first and third quartiles, the mean is displayed. Statistical analysis compared H2B S6ph between different cell lines and HCT116 cells, ***p  ≤  0.001. (*d*) HCT116 and U2OS cells in late anaphase were analysed by immunofluorescence for the intracellular localization of H2B S6ph and Aurora B, which was detected at the chromatin or the central spindle. Scale bar = 10 µm. (*e*) The indicated cell lines were analysed for the localization of Aurora B and the occurrence of H2B S6ph. The graph shows a statistical analysis where the diameter of the circles corresponds to the percentage frequency, while the outer ring indicates the value of the upper standard deviation.

While H2B S6ph is lost after early anaphase in diploid cells [34], we wondered whether CIN observed in a number of tumour cells is associated with the deregulation of H2B S6ph. Screening of a panel of different tumour cells and also control diploid cells for mitotic H2B S6ph showed frequent phosphorylation during late anaphase for cell lines such as HT-29 (colorectal adenocarcinoma) and U2OS (osteosarcoma), while other tumour cells showed proper timing of this phosphorylation (figure 7*a*). H2B S6ph observed during late anaphase was particularly enriched at chromosomal aberrations of some cell lines, as displayed for HT-29 or U2OS cells (figure 7*b*) and quantified for a larger cell panel (figure 7*c*).

As Aurora B is a key factor maintaining H2B S6ph during anaphase, we asked whether this persistent H2B S6ph might be attributable to lacking translocation of Aurora B from chromatin to the central spindle during anaphase. Co-staining of H2B S6ph and Aurora B in different cell types revealed that the appearance of H2B S6ph during late anaphase frequently correlated with chromatin localization of Aurora B (figure 7*d*). We then asked whether this correlation also occurs in tumour cells displaying frequent H2B S6ph during late anaphase. All cell lines analysed, regardless of their high (RPE-1, HCT116) or low (U2OS, HT-29) chromosomal stability, showed a striking correlation between appropriate H2B S6 dephosphorylation in late anaphase and Aurora B localization at the central spindle (figure 7*e*).

**Figure 8.**
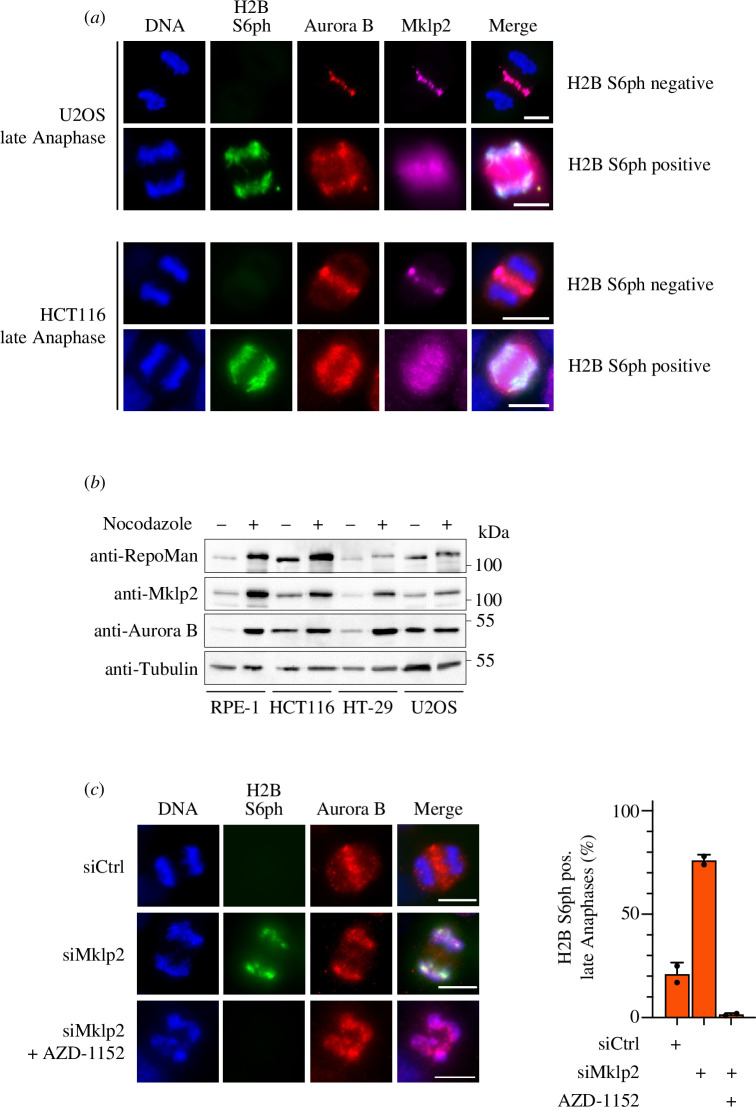
Effects of Mklp2 levels on Aurora B localization and H2B S6ph. (*a*) Mitotic HCT116 and U2OS cells were analysed for the occurrence and localization of Mklp2, Aurora B and H2B S6ph in late anaphase. Representative examples of the different phenotypes are displayed. (*b*) The indicated cell lines were treated with nocodazole or DMSO for 16 h and whole cell extracts were prepared, followed by Western blot analysis using the indicated antibodies. (*c*) HCT116 cells were treated with siRNAs targeting Mklp2 and treated for 15 min with AZD-1152 as shown. The occurrence of H2B S6ph and the localization of Aurora B was analysed in late anaphase by indirect immunofluorescence, scale bar = 10 µm. The right part shows a quantitative analysis of the experiment displayed in (*c*). Error bars indicate standard deviations from two independent biological replicates.

The transport of Aurora B from chromatin to the central spindle critically depends on the motor protein Mklp2 [38–40] and accordingly we always detected the colocalization of Aurora B with this motor protein in different cell lines (figure 8*a*). Aberrant expression of the Mklp2-encoding *KIF20A* gene and the RepoMan-encoding *CDCA2* gene have been observed in a variety of cancers and significantly correlates with survival outcomes [41–44]. Interestingly, it was the genetically unstable HT-29 and U2OS cells that exhibited a significant defect in mitotic upregulation of Mklp2 and RepoMan proteins (figure 8*b*). These results raise the possibility that relative expression levels of the Mklp2 motor protein might indirectly also affect H2B S6ph. Downregulation of Mklp2 expression by siRNAs prevented chromatin removal of Aurora B and consequently led to persistent H2B S6ph during late anaphase in an Aurora B kinase-dependent manner (figure 8*c*), revealing another indirect downstream target of this motor protein.

## 3. Discussion

The duration, amplitude, threshold and localization of mitotic phosphorylations require precise orchestration for the stepwise sequence of mitotic phases to proceed [45]. This is not only dependent on an antagonistic interplay between kinases and phosphatases, but in addition on cooperative cross-regulation between these enzyme groups [46,47]. An example for kinase-directed control of phosphatase function is provided by RepoMan. While RepoMan’s ability to interact with chromatin is reduced by Aurora B-mediated phosphorylation of S893 [23], interaction with PP1 is diminished by CDK1/cyclin B-mediated phosphorylation at T412 and probably further sites at S400 and T419 [24,25]. On the other hand, phosphatases can also restrict kinase activity by removal of phosphates from the activation loop [48,49], or they even trigger kinase activity as exemplified by CDC25A, which removes an inhibitory phosphorylation from CDK1 [50].

This study shows that the PP1/RepoMan complex coregulates the restriction of localization, timing and intensity of mitotic H2B S6ph to the inner centromere during the early stages of mitosis and its ultimate removal in later stages of cell division. Although this complex mediates dephosphorylation of various histone modifications including H3 T3, H3 S10 [26,35] and H2B S6 (this study), the removal of phosphates from H2B S6 occurs first. At the phosphatase level, these different kinetics could be explained, by the involvement of different and yet unknown accessory proteins and mechanisms. An Aurora B-independent mechanism restricting the activity of the PP1/RepoMan complex is the CDK1/cyclin B-mediated phosphorylation of RepoMan at S400, T412 and T419 [25,26]. Also, these modifications preclude binding of PP1γ and probably further phosphatase subunits [24]. Accordingly, H2B S6ph during late anaphase was also observed in cells with Aurora B localization at the central spindle (figure 7*e*). Although PP1/RepoMan is essential for the dephosphorylation of the histone phosphorylations studied here, the involvement of additional scaffold proteins and phosphatases cannot be formally excluded. It is not clear to which extent PP1α activity will be affected by the elimination of RepoMan, as this PP1 subunit also interacts with other targeting subunits such as nuclear inhibitor of PP1 (NIPP1) and Rap1-interacting factor 1 (RIF1) [51,52]. Furthermore, differences in the kinetics of histone phosphorylations could also be attributable to distinct activation periods of the relevant kinases. While the activity of the H2B S6 phosphorylating CDK1/cyclin B complex terminates at the end of metaphase [53,54], the activity of the H3 S10-phosphorylating Aurora B kinase decreases later and remains even after its relocation to the central spindle [55].

It is currently unclear whether the accumulation of H2B S6ph on chromosomal aberrations is a consequence of the mislocalization of phosphorylating and dephosphorylating enzyme complexes, or alternatively contributes to a (patho)physiological process. Functional roles for enriched histone phosphorylations on lagging chromosomes were found for H3.3 S31ph, which triggers p53-mediated cell cycle arrest [56]. Also, an excess of H3 T118ph results in increased numbers of lagging chromosomes [57]. Therefore, it would be important to systematically identify the PTMs on lagging chromosomes and to reveal their potential functional significance. Multiple reasons can cause dysregulated histone phosphorylation on lagging chromosomes. These include not only misregulation of the phosphatases and kinases involved, but also—as shown, to our knowledge for the first time in this study—changes in levels of the Mklp2 protein and its dynamic regulation. It will therefore be interesting to investigate the relative contribution and consequences of dysregulation of Mklp2 and probably further motor proteins on histone modifications and CIN in the future.

## 4. Material and methods

### 4.1. Antibodies, primers and plasmids

This information is given in the electronic supplementary material, table S1.

### 4.2. Cell culture

HCT116, HeLa, U2OS, LN-229, Caco-2, T98G, mouse embryonic fibroblasts, 293T and MCF7 cells were grown in Dulbecco’s modified eagle medium (DMEM), SW620 and HT-29 cells in RPMI and RPE-1 cells in DMEM/F12. All media were supplemented with 10% fetal calf serum (FCS) and 100 U ml^-1^ penicillin and 100 µg ml^-1^ streptomycin. The cells were cultured at 37°C and 5% CO_2_ in a humidified atmosphere.

### 4.3. Cell synchronization and mitotic block

HCT116 cells were synchronized with a single thymidine block. Thymidine was added to a concentration of 2 mM for 18 h. Cells were then washed three times with warm phosphate-buffered saline (PBS) and released into fresh medium and analysed 10 h later. HCT116 were arrested in mitosis by addition of 100 ng ml^-1^ nocodazole for 16 h. HeLa cells were synchronized by addition of RO-3306 to a concentration of 9 µM for 18 h. Cells were then washed twice with warm PBS and released into fresh medium and analysed 2 h later.

### 4.4. Cell transfection

Cells were seeded 1 day prior to transfection to result in 50% confluence at the time point of transfection using polyethylenimine (PEI). Shortly, DNA and PEI were diluted in separate tubes and then combined in a ratio of 3 µg PEI per 1 µg of DNA as described [58]. Cells were washed and covered with antibiotic-free medium, and the transfection mixture was added dropwise to the cells. After 4 h, the medium was aspirated and replaced with complete medium. Transfections with siRNAs were performed with Lipofectamine 3000 according to the manufacturer’s protocol.

### 4.5. Chromosome spreads

Cells were arrested in prometaphase with 100 ng ml^-1^ nocodazole for 4 h or overnight. Mitotic cells were collected by shaking-off and centrifuged at 300× g for 5 min. Cells were washed with PBS and swollen in 0.8% (w/v) sodium citrate for 10 min at room temperature (RT). A total of 50 000 cells were spun on glass slides with a Cellspin III (Tharmac) cytocentrifuge. Cells were then fixed in 3.7% (v/v) formaldehyde/PBS for 10 min at RT and washed in KCM buffer (120 mM KCl, 20 mM NaCl, 10 mM TRIS/HCl pH 8.0, 0.5 mM EDTA and 0.1% (v/v) Triton X-100) for 30 min at RT. The slides were used for staining of DNA and proteins as described below.

### 4.6. Immunofluorescence staining and microscopy

Cells were grown on 18 mm glass coverslips. Cells were fixed on coverslips with 3.7% formaldehyde/PBS for 10 min at RT and treated with permeabilization buffer (3% (w/v) bovine serum albumin (BSA)/0.3% (v/v) Triton X-100 in PBS) for 30 min at RT. Coverslips were incubated with primary antibodies in permeabilization buffer overnight in a humidified chamber at 4°C. After washing coverslips several times with permeabilization buffer, they were incubated with dye-coupled appropriate secondary antibodies in a humidified chamber for 1 h at RT in permeabilization buffer. DNA was counterstained with 1 µg ml^-1^ Hoechst 33 342 and washed three times with PBS. Coverslips were mounted on standard glass slides with Mowiol mounting medium. Cells were analysed on an Eclipse TE2000-E inverted fluorescence microscope (Nikon) equipped with a cooled pE-300 light source, an ORCA Spark CMOS camera (Hamamatsu) and a T-RCP Controller (Nikon). Confocal microscopy was performed on an Aurox Unity spinning disk confocal microscope. Images were recorded with NIS Elements 3.10 or the Aurox Unity app and processed with Fiji (ImageJ 2.1.0/1.53c) and Microvolution. Mitotic figures were classified manually based on morphological cues. An incipient but incomplete separation of chromatids was considered as early anaphase, whereas a clear gap between chromatids was defined as late anaphase. Cells showing a signal distinctly above the staining background were counted as positive, all cell biological analyses were conducted in a blinded manner. For visualization, images were processed with Fiji. Only uniform, linear brightness and contrast adjustments were used.

### 4.7. GFP-Trap^®^ experiments

Human 293T cells were transfected to express the EGFP-RepoMan fusion protein. The next day, cells were lysed in cold lysis buffer (20 mM Tris/HCl, pH 7.5, 150 mM NaCl, 10% (v/v) glycerol and 1% (v/v) IGEPAL C-630) supplemented with protease and phosphatase inhibitors (4 µg ml^-1^ aprotinin, 4 µg ml^-1^ leupeptin, 0.5 mM phenylmethylsulfonylfluoride (PMSF), 20 mM NaF and 1 mM Na_3_VO_4_). One aliquot of the lysate was used for the input control, while the remaining material was diluted with GFP-Trap^®^ dilution buffer (10 mM Tris/HCl, pH 7.5, 150 mM NaCl and 0.5 mM EDTA), followed by incubation with 10 µl of GFP-Trap^®^ agarose beads for 2 h. Beads were pelleted and the supernatant was removed. Beads were washed four times with cold dilution buffer and proteins were eluted by boiling in 1× sodium dodecyl sulfate (SDS) sample buffer, followed by further analysis by Western blotting.

### 4.8. Western blotting

Cell extracts were prepared with cold RIPA buffer (10 mM TRIS/HCl pH 8.0, 1 mM EDTA, 0.5 mM EGTA, 1% (v/v) Triton X-100, 0.1% (w/v) Na-Deoxocholate, 0.1% (w/v) SDS and 140 mM NaCl) supplemented with protease and phosphatase inhibitors (4 µg ml^-1^ aprotinin, 4 µg ml^-1^ leupeptin, 0.5 mM PMSF, 20 mM NaF and 1 mM Na_3_VO_4_). Extracts were sonicated using a Branson Sonifier Minitip, and protein concentrations were determined by bicinchoninic acid (BCA) protein quantification. Samples were prepared for electrophoresis by boiling with SDS Laemmli sample buffer. Equal amounts of protein lysate were separated by SDS–polyacrylamide gel electrophoresis (PAGE) and transferred to a polyvinylidene fluoride (PVDF) membrane. Membranes were probed with primary antibodies overnight at 4°C on a rotator. After washing four times with Tris buffered saline with Tween (TBS-T) (137 mM NaCl, 2.7 mM KCl, 19 mM TRIS, pH 7.4 and 0.1% (v/v) Tween 20), membranes were probed with appropriate secondary antibodies coupled to horseradish peroxidase. After washing four more times, the signal was detected with Western Lightning enhanced chemiluminescence (ECL) (Perkin Elmer) using a ChemiDoc XRS (Bio-Rad).

### 4.9. Coomassie staining and enzyme linked immunosorbent assay

Recombinant proteins were separated by SDS–PAGE and subsequently analysed by Coomassie staining to analyse protein size, concentration and purity. Gels were washed with deionized MilliQ water and incubated with Coomassie staining solution (45% (v/v) methanol, 10% (v/v) acetic acid and 0.25% (w/v) Coomassie Brilliant Blue-G250) at RT for at least 1 h. Stained gels were washed again and transferred to the destaining solution (30% (v/v) methanol and 20% (v/v) acetic acid) for at least 2 h at RT.

Enzyme linked immunosorbent assay (ELISA) was performed with NeutrAvidin-coated 96-well plates (Pierce) and biotinylated peptides. Pre-blocked plates were washed three times with ELISA wash buffer (TBS-T with 0.1% (w/v) BSA). Peptides were dissolved in PBS, and 2.5 μg peptide/well was added, binding occurred at RT for 2 h. Unbound peptides were removed by washing several times with ELISA wash buffer, followed by incubation with different dilutions of test antibodies for 20 min. After three washes, secondary horseradish peroxidase (HRP)-conjugated antibodies were added for 20 min. Wells were washed again three times, and substrate solution (110 mM sodium acetate (pH 5.5), 0.1% (v/v) H_2_O_2_ and 0.1 μg ml^-1^ tetramethylbenzidin (TMB)) was added for 5 min. After the addition of one volume stop solution (10% (v/v) H_2_SO_4_) antibody binding was quantified by determination of emission at 450 nm.

4.10. Quantitative reverse transcription polymerase chain reaction

Cells were collected by trypsinization after washing with PBS. Cells were pelleted and total RNA was extracted using a NucleoSpin RNA kit (Macherey-Nagel) following the manufacturer’s protocol. The eluted RNA was quantified with an Eppendorf 6131 photometer and 500 ng RNA were reverse transcribed (RT) with SuperScript II RT (Invitrogen) and oligo-dT primers or PrimeScript RT Master Mix (Takara). Generated cDNA was diluted 1:5 using RNase-free water. Equal volumes of cDNA were used as a template for amplification with SYBR Green ROX Mix (Thermo). Reactions were performed in triplicates in 96-well polymerase chain reaction (PCR) plates on an OneStep Plus (Applied Biosystems) cycler with SYBR Green as the reporter and ROX as the passive reference. Quantitative PCR data were analysed using the ∆∆Ct method as described [59]. Shortly, the *C*_t_ values of the target gene were normalized to the *C*_t_ values of a housekeeping gene for the treated and control samples, respectively. The treated sample was then normalized to the untreated sample.

### 4.11. *In vitro* kinase and phosphatase assays

*In vitro* kinase assays were performed with recombinant proteins. 0.5 µg of CDK1 and cyclin B1 and 2 µg H2B were added to a kinase reaction mix (6 mM HEPES, pH 7.5, 3 mM MgCl_2_, 3 mM MnCl_2_, 1.2 mM DTT, 5% (v/v) glycerol and 20 μM ATP) and incubated at 37°C for 30 min. The phosphorylation was stopped by heat inactivation at 95°C for 10 min. 0.2 µg GST or PP1 enzyme were added and mixtures were incubated for another 30 min at 37°C. Reactions were stopped by the addition of SDS sample buffer and samples were further analysed by Western blotting or Coomassie staining.

### 4.12. Monoclonal antibodies detecting H2B S6ph

Rat monoclonal antibodies against H2B S6 phosphorylation site were generated by immunization of Lou/c rats with ovalbumin-coupled peptides (aa 1–11, PEPAKpSAPAPK) comprising phosphorylated S6 (Peps4LS, Heidelberg). Animals were injected subcutaneously (s.c.) and intraperitoneally (i.p.) with 40 μg peptide, 5 nmol CpG 2006 (TIB MOLBIOL, Berlin, Germany) and an equal volume of incomplete Freund’s adjuvant. After six weeks interval, a final boost with the phosphorylated peptide and CpG 2006 was given i.p. and s.c. 3 days before fusion. Fusions of the myeloma cell line P3X63–Ag8.653 with the rat immune spleen cells were performed according to standard procedures. Hybridoma supernatants were screened for binding to biotinylated phosphopeptides coupled to streptavidin beads (PolyAn Red4 Multiplex Beads, Berlin, Germany) in a multiplex flow cytometry immunoassay (iQue, Intellicyt; Sartorius, Göttingen, Germany). Specificity was confirmed by negative screening on biotinylated non-phosphorylated peptides. Positive supernatants were further validated by Western blot analysis. Hybridoma cells from selected supernatant 1D4 were subcloned twice by limiting dilution to obtain a stable monoclonal cell clone. Experiments in this work were performed with hybridoma supernatant H2B6P 1D4 (rat IgG2b).

### 4.13. Quantification and statistical analysis

Image Lab 6.0.1 (Bio-Rad) and ImageJ/Fiji 1.53c software were used for image processing and densitometric analysis of Western blot data. GraphPad Prism 9 (GraphPad Software, La Jolla, CA, USA) was used to perform statistical analysis and visualization. Unless otherwise noted, diagrams show the mean and error bars indicate standard deviation of at least three biological replicates. When the mathematical prerequisites are met (*n* ≥ 3) and statistically significant differences exist, *p*-values were provided in the figures (*p  ≤  0.05, **p   ≤  0.01, ***p  ≤  0.001). All raw data for statistical analysis are shown in the electronic supplementary material, table S2. Schematic figures were created using BioRender.com.

## Data Availability

Electronic supplementary material is available online [60].
